# The moment of youth: learning from youth-led research on sexual and reproductive health and rights (SRHR) in Malawian and Zambian settings

**DOI:** 10.1186/s40900-025-00822-z

**Published:** 2026-01-12

**Authors:** Tasneem Kakal, Charlotte van Tuijl, Kettie Mikwala, James Zimba Junior

**Affiliations:** 1 Department of Global health, KIT Institute, Amsterdam, The Netherlands; 2 Erasmus School of Health Policy and Management, Erasmus University Rotterdam, Rotterdam, The Netherlands; 3 Erasmus School of Social and Behavioural Sciences, Erasmus University Rotterdam, Rotterdam, The Netherlands; 4 Independent Consultant, Zomba, Malawi; 5 Independent Consultant, Lusaka, Zambia

**Keywords:** Youth-led research, Malawi, Zambia, SRHR, Youth

## Abstract

**Background:**

Research about young people in lower- and middle-income countries is seldom done by young people themselves. Engaging youth in designing and conducting research that concerns their well-being can improve their skills and make the findings more relevant to young people’s needs. This commentary outlines five lessons learned in the process of conducting a youth-led research study in Malawi and Zambia on youth sexual and reproductive health and rights for the Break Free! programme. Reflections on the challenges and successes encountered by the research team during this study were documented via notes. A reference group of youth advocates was set up to guide the study, and an evaluation was conducted with them about their experience. Data from both these sources were thematically organised and discussed between the research team.

**Lessons learned:**

The commentary outlined that meaningfully involving youth in the study methods, not only as participants but also with a youth-led research team and a youth reference group enhanced the quality of study findings. The combination of seasoned youth researchers working with emerging youth researchers from prior collaborations laid a good foundation to implement the study and was identified as a success factor. Fostering a positive working culture that centred peer-to-peer support and the expertise of researchers in and from the ‘Global South’ was a key success in having a fair partnership that shifted power. However, time taken for mentoring, and knowledge exchange, between youth researchers and with the youth reference group was under-estimated and under-budgeted and an adaptable approach to time and resources allowed the research team to address these challenges. Lastly, the support of an established institution was instrumental in the conceptualization and execution of the study and functioned as a pre-condition for success.

**Conclusion:**

The lessons outlined in the commentary highlights the added value of engaging youth in all stages of the research cycle and can offer food for thought for future researchers looking to adopt this approach. While youth-led research may not be suited to every context, its principles of equity and participation can transform both the young participants and the research process.

## Abstract (de)

Research about young people is often not done by young people themselves. But when young people help design and do research about their own lives, they can learn useful skills. It makes the results more relevant for young people’s needs.

Four youth researchers led a study and shared five lessons they learned:

1. Having a team including young people with different levels of research experience meant they could help and learn from each other.
2. This study was possible because a well-known organisation supported the team from the start by giving money, resources, and trust to try a new way of doing research.
3. Meaningfully involving young people in different parts of the research made the results more useful. Youth took part as participants, advisors and led the research. This made the questions easier to understand for young people and gave better insight into their perspectives.
4. Not all young people are the same. In an international team, people have different backgrounds, privileges and access to opportunities. The team worked well because members with more opportunities made space for others to share their views.
5. It was important to plan for enough time, money, and flexibility to carry out the research and meaningfully engage young people. This made it possible for youth researchers to support and mentor each other.

In conclusion, youth-led research can strengthen young people’s ability to take part in decisions about their lives. It can also offer more relevant ways of understanding the issues young people face.

## Background

The achievement of the Sustainable Development Goals relies on the meaningful engagement of young people in decision-making processes that impact their lives [1]. Even though more than half of the population on the African continent are youth (15–35 years), they are not adequately engaged in decision-making processes [2]. Engaging young people in youth-led research, where young people lead all stages of research — from design to dissemination — is a pathway to produce evidence that can inform policy processes that affect their lives, build youth capacities, increase the visibility of their perspectives, and stimulate youth leadership in the research process [3, 4]. Indeed, young people are eager to contribute to the development of youth-related policies and research [5, 6].

The many benefits of youth-led research are also accompanied by certain challenges. Lack of capacity among youth researchers, lack of trust from adults in the ability of young people, or views that young people might not be objective when collecting data from their peers, are factors which might hamper the uptake of youth-led research [4].

Most of the evidence on youth-led research originates from the field of child studies and social work, particularly from North America. There is limited evidence in other fields and from low- and middle-income countries (LMICs). There have been a few recent efforts to conduct youth-led research. For instance, Restless Development worked with a team of twelve youth researchers (18–29 years) to conduct a youth-led research study on the future of education and the impact of the pandemic on youth across 70 countries [7]. Other efforts have been undertaken by UNESCO [8], Restless Development [9] and International Planned Parenthood Federation [10]. Nevertheless, most studies focus on forms of youth participatory action research [11–14]. This commentary aims to address this gap and contribute to the nascent but growing use of youth-led research methodologies. The commentary reflects on the experience of conducting youth-led research in Malawi and Zambia, documenting the lessons learned and situating its value in the field of adolescent and youth sexual and reproductive health and rights (SRHR).

Responding to the call of Ozer et al. (2018) [15] to clearly document the approaches and challenges of youth-led research, this commentary outlines five key lessons learned on conducting youth-led research, including pre-conditions and factors for success.

## Methodology

### Context of collaboration and programme description

We wish to provide context to this commentary by briefly outlining the study that brought us to collaborate. The youth-led study we are reflecting on in this commentary was conducted for the Break Free! programme (2021–2025), which aims to strengthen young people’s SRHR and promote gender equality in nine African countries. The programme is implemented by the Break Free! Alliance led by Plan International (Netherlands) in the lead, together with SRHR Africa Trust (SAT) and Forum for African Women Educationalists. KIT Institute (KIT), formerly known as KIT Royal Tropical Institute is the research partner. ‘YouthWyze’ is a multi-level intervention implemented under the programme. Its online component consists of an active social media presence with a Facebook page providing SRHR information, while the offline component includes youth clubs that provide SRHR information and youth-friendly outreach SRH services. This study sought to explore how young people between 15 and 24 years access SRHR information and services through online and offline modes, with a primary focus on YouthWyze. The study was conducted in 2021–2022.

The research team consisted of TK (research lead) and CT (co-lead) based at KIT Institute, and KM and JZ (also referred to as in-country researchers) based in Malawi and Zambia. TK and CT, had been working with the Break Free! programme since 2020 and conceptualised the study with the backing of a senior researcher, who led KIT’s on-going collaboration with the Break Free! Alliance. The senior researcher is also experienced in research on youth SRHR and endorses the meaningful involvement of youth in research. TK and CT conceptualised the youth-led study design and worked with the Break Free! Alliance to form a youth reference group (henceforth referred to as reference group) [16, 17], that supported the research process and guided the study. The reference group consisted of five SRHR advocates from Malawi and Zambia (under 30 years) who were involved in the intervention. They contributed to defining the study design, co-developed study tools, and validated study findings. The group included two SAT employees working as ‘Youth Officers’ – responsible for implementing the intervention ‘YouthWyze’ under the Break Free! programme. The Youth Officers supported the study since its inception. The rationale for setting up a reference group was to centre the knowledge and experiences of youth advocates throughout the research cycle and promote accountability between the reference group and the research team [18].

The study included focus group discussions (*n* = 8 groups) with young people, in-depth interviews with health workers (*n* = 8), youth (*n* = 6) and key informants (*n* = 4) in the districts of Machinga and Lilongwe (Malawi), Chipata and Kasenengwa (Zambia) and Lusaka city (Zambia). Alongside, an online survey (*n* = 107) was conducted with YouthWyze users. We also organised a ‘Future Forward’ workshop with youth advocates, including the reference group members and some study participants, in each country to discuss the preliminary findings and recommendations. Foresight methods [19] were used in the workshop to brainstorm on the future of SRH services. The objective was to use young people’s imagination as data, fuel creativity and use youth-friendly methods of inquiry (such as drawing, dreaming and interactive activities).

### Methods

The five lessons outlined draw from the challenges and successes that we, as the research team, identified during the process of conducting this youth-led study. These lessons emerged using two methods. The first included our personal reflections elicited during regular check-in meetings through the course of the study, which were documented via minutes by TK and CT and were shared with KM and JZ consistently. The second was an evaluation with the reference group. This included the feedback received at the end of each working session between the research team and the reference group (5 sessions in total). In addition, a survey was conducted with the reference group at mid- and end-term to get feedback on their experience and the quality of their participation.

These reflections were documented via notes during the research process and TK and CT organized these into main themes (which are presented below) during a writing workshop at KIT Institute. This documentation was shared with KM and JZ who provided nuance and made additions. TK and CT took the lead in conceptualization, analysis and writing of the commentary. Moreover, this commentary was conceptualized once the study ended due to interest at KIT to understand and document this unique approach. To ensure that JZ and KM remained involved to some extent while still respecting their time, they were asked for their reflections on the main themes and the drafts.

The reflections in this commentary were reviewed by colleagues at KIT and were presented at an internal learning day in the organisation. Constructive criticism from that session was incorporated into the commentary.

We believe it is important to situate our approach and social location when discussing youth-led research [20]. TK and CT are Indian and Dutch/Luxembourgish respectively while KM and JZ are from Malawi and Zambia respectively. TK and CT were based at KIT in the Netherlands at the time of the study. They are experienced in conducting mixed-methods research on SRHR across LMICs, including in Malawi and Zambia. KM and JZ have worked with TK on prior mixed-methods studies as research assistants, with the former working with TK on multiple projects. At the time of the study, the research team members were between 25 and 29 years. TK, CT and KM identify as women and JZ as a man. The underlying principle of the study discussed between all of us strived to centre meaningful and inclusive involvement of youth [21]. TK and CT were cognizant of promoting fair partnerships, particularly in the context of decolonising research and take an intersectional approach during study conceptualisation. We recognise that ‘youth’ is not a homogenous group, evidenced by the diversity of privilege, experiences and identities between us as the research team, and the study participants we work with. Throughout the study, we have strived to be accountable to our biases through open communication.

## Lessons learned

In the following section, we reflect on the challenges and successes that were identified during the process of conducting this youth-led study.

### Putting together a team: combining seasoned and emerging researchers

TK and CT began the process of putting together a study to learn from the implementation of the YouthWyze intervention with the two Youth Officers in Malawi and Zambia. At this stage of study conceptualisation, all individuals involved were youth. In line with KIT’s modus operandi of partnering with research institutes and researchers based in the research areas, and given the study design of having youth researchers conduct the study, TK and CT sought to contract candidates below 30 years. They sought candidates from Malawi and Zambia, who were knowledgeable about SRHR and had some experience in conducting and managing research. TK and CT chose this age cut-off as it would allow for candidates who had accumulated some research experience, but this age also remained close to the target group of the study which was between 15 and 24 years. This age cut-off also followed existing youth-led research models [7]. Nevertheless, it was challenging to find candidates who met all the eligibility criteria because young people, particularly in LMICs, are often only involved in data collection activities. To address this, TK intentionally built on established collaborations from previous youth-centred research projects in Malawi and Zambia, where she had trained young research assistants in ethics, data collection and the basics of data analysis and writing [4]. Drawing on these relationships, JZ and KM joined this study. When reflecting on the successes, we believe that these prior collaborations translated to a stronger foundation of mutual trust and shared research skills. This prior capacity-building contributed to a smoother and more equitable working relationship within this youth-led study design.

The combination of seasoned and emerging researchers comprising the research team worked well and created complementarity and room for mutual growth. While KM and JZ had more insight into the local contexts and had some experience doing data collection and research in the study areas, TK and CT were seasoned researchers and brought more project and broader research experience, while being backed by an established institution. In addition, there were differences between the capacities of the in-country researchers, KM and JZ and between TK and CT. Since KM based in Malawi had more experience in organising data collection logistics and conducting research, she aided the researcher JZ in Zambia and shared anticipated challenges with the researchers at KIT. The in-country researchers were also supported by the Youth Officers in each country. TK had considerable experience conducting (and leading) mixed-methods research, had done research in Malawi and Zambia, and supported CT’s growing research and work experience, while CT brought her expertise on meaningful involvement of young people to the team. When the team encountered difficulties, we worked with the senior researcher who created a safe space for learning, guidance and resolution. Hence, the collaboration with the senior researcher was crucial to the success of the study. This was facilitated by the senior researcher’s trust and their oversight ‘from a distance’. The senior researcher supported us in aspects of financial management and contributed to report writing.

### Adaptability is required – in approach, time and resources

Through the process of conducting this research study and reflecting on it, it became clear that youth-led research required adaptability and flexibility with regards to approach, time and budget. While some aspects were accounted for in advance, other challenges emerged along the way.

We recognised that members of the reference group did not have experience designing study tools or conducting research. To account for this, we planned for and organised multiple joint-virtual working sessions with the reference group where we introduced research concepts. We intentionally did this, so as to build capacities mutually among the team. Along with the youth reference group, the research team jointly developed a draft of the tools, with KM and JZ, the in-country researchers, playing an important role in steering this process. While this worked well virtually, during a reflection on the process with the reference group members, it became evident that in-person interaction could have greatly improved the quality and experience of the work. The reference group members underscored the need for physical meetings to connect and exchange knowledge. Hence, this was one of the motivating factors in holding an in-person data analysis workshop amongst us. The goal was to ensure each research team member was comfortable and equipped with the skills needed for research analysis and writing.

Alongside research experience, TK and CT also recognized that younger researchers often have limited opportunities to work on project management, including budgeting for research projects. To mitigate any ensuing challenges for this project, KM and JZ worked in close collaboration with TK and CT at KIT and extensively discussed the research preparations. Despite anticipating this need, over time, TK and CT learned that these check-ins required more resources than planned for and were not adequately built into the budget. The logistics of budget organisation and invoicing took up more time than expected, and the guidance of an experienced senior researcher proved vital in the process. Having a flexible budget allowed us to spend more time, and adapt the approach of working together as challenges emerged.

### Enhanced quality and relevance of the study design and findings

There was an improvement in the quality and relevance of the findings due to the involvement and leadership of young people in the research process. This is illustrated by several instances. During the process of developing the research tools, the reference group’s contributions led to the survey using simpler language and playful elements such as emojis. All reference group members felt that the participatory approach of tool development improved the study tools. In the mid-term survey, a member shared, “I have learnt to listen and incorporate other people’s views in research as it is more representative of the target population when multiple perspectives are taken into account when coming up with research tools”. At mid-term, another member echoed this sentiment, “It’s good to make the development of research tools consultative and participatory, and inclusion of young people on the ground […] brings in a different insight”.

Having youth researchers interview youth participants is a strategy whose benefits (e.g. increased relatability) have been well documented [20]. For instance, KM felt that her position as a young person allowed the participants to easily communicate with her, making the conversation feel like one between friends, mitigating some of the power dynamics that ensue between adult researchers and youth [22]. Particularly with regards to topics related to SRHR and sexuality, young people communicate differently with other young people, in a non-judgemental manner [10]. More importantly, it was advantageous to have younger researchers leading the study as they were users of social media, familiar with online styles of communication, and could understand how social media could enhance the SRHR of young people.

During the ‘Future Forward’ workshop, we also validated the findings with youth advocates by presenting the findings and inviting youth advocates to comment on their relevance and share their insights [19]. The discussions in the workshops and in the reference group – based on lived experiences – allowed us to identify ‘blind spots’, provide nuance to the findings and input into the recommendations. Diversifying the language used by the YouthWyze Facebook page was a recommendation by young people in Malawi and Zambia and this was further discussed in the workshops. Workshop participants recognised the challenges that would ensue – i.e. how to choose which local languages to use, either to post or to translate the posts. In a similar vein, a youth advocate recommended using slang words in the posts to increase relatability, to which a reference group member flagged another concern – that this would reinforce parental concerns that the internet was an unsafe space. The reference group played an active role in discussing the findings and they put forth concrete ideas of disseminating them in a youth-friendly manner. Due to their suggestion to have colourful visuals shared through the YouthWyze Facebook page, TK and CT developed short visual summaries for online and offline use. Moreover, given that young people were involved at all stages of the research, the Break Free! Alliance valued the recommendations and started exploring supplementary options such as the radio to reach youth in rural areas.

### Trust among different stakeholders

There were several stakeholders in this study, who were invested in its process and the final findings. This included KIT leading the study, the Break Free! Alliance, the research participants which included both adults and youth, the reference group and the research team. KIT is a reputed knowledge institute with extensive experience conducting research in LMICs. This meant that both researchers from KIT and the contracted in-country researchers had the backing of an ‘adult’ institution towards partners of the Break Free! Alliance and community-level stakeholders. Plan International (Netherlands) had previously collaborated with KIT, including with the KIT research team (particularly with the senior researcher and TK). This ensured trust in KIT, and this partnership provided the necessary financial resources to implement the study approach and fairly compensate the reference group and in-country researchers for their contributions. Moreover, for Break Free!, including SAT (that implements YouthWyze), youth empowerment is integral to their organisational strategy, evident from the presence of Youth Officers representing SAT during the study. All these factors built an environment of mutual trust and respect in the research team and in the study approach [10]. Interestingly, a young male participant in Lilongwe displayed scepticism towards the study design, but quickly took a positive perspective at the end of his interview: “I never thought a young person like you can be doing this type of work. It’s really amazing. I am impressed”.

### Creating a culture of working together and shifting power

There was a strong emphasis on creating a positive working culture, where young people could be vulnerable, develop themselves professionally and felt comfortable to hold each other accountable. In the spirit of creating a fair partnership, TK and CT prioritised the task and attempted to do this while acknowledging the different skills, genders, social locations, and levels of power within and between the research team and reference group members.

This included setting clear expectations, inviting all reference group members to share their professional development goals at the beginning of this study [6] and emphasising the adage: ‘there are no stupid questions’ [20]. The reference group members valued learning about virtual collaboration and working in a team. At end-term, one reference group member reflected, “I learnt that teamwork is key to achieving anything…I did not think I could manage to do this YRG [youth reference group] virtually but I learnt that [I] am a quick learner and can easily adapt to technology”. In the mid- and end-term survey conducted with them, they described their participation with words such as ‘meaningful’, ‘team’, ‘participation’, ‘engaging’, ‘mind opening’ and ‘insightful’. In the mid-term and end-term survey, all reference group members strongly agreed that the youth reference group was participatory, that they could give their opinion and be heard in the group, and that every member respected each other. They reported having learned a lot owing to their end-to-end involvement in the research process from design to dissemination. Since they were involved in YouthWyze, the study design limited their engagement during the data analysis phase to the validation of the findings to ensure independence of results. However, the analysis took longer than expected which led to a long absence of communication towards the reference group during this phase. We believe this broke the momentum, due to which their participation in planned activities waned. This highlighted the importance of consistent engagement and outlining a role for the reference group during the analysis phase could have mitigated this.

Given that TK and CT had contracted the in-country researchers, there was bound to be a power dynamic that emerged between the contractor and contracted, especially given that a ‘Global North’ institution was contracting individuals of the ‘Global South’ [23]. Hence, they took the lead to explicitly acknowledge the power dynamics at play during the research team’s meetings. TK and CT took deliberative efforts in an attempt to shift power [15]. TK and CT strived to put KM and JZ’s expertise in the forefront during the reference group meetings as well as towards the Break Free! Alliance members. KM and JZ were consistently sought for their advice by TK and CT and co-presented the research findings to the Alliance. In fact, JZ felt that he had complete ownership of this project as he was fully involved in all aspects of the research and was the focal person to answer questions about the study for Break Free! Zambia. CT also supported KM in Malawi to present the study findings at a national conference. Through the research cycle, it also emerged that the KM and JZ could support each other, and they expressed that they had grown personally and professionally during this study. They discussed their concerns prior to meeting TK and CT in online meetings. TK and CT observed that the longer the in-country researchers worked together, the more confident they felt to ‘voice’ their concerns as a unit (for instance, on timely disbursements of funds), virtually and even more so during the in-person workshop. This is also telling because attempts to shift power, while important, cannot fully dismantle the power dynamics that ensue in a contractual collaboration between the ‘Global North’ and ‘Global South’ [23]. This also points to the added value of working in teams, instead of only hiring one youth researcher who works in a silo.

## Conclusions

We have outlined five lessons, drawing from the challenges and successes encountered during the study. The five lessons outlined can be framed as two essential preconditions and three critical factors for success that can guide other organisations in their youth-led research efforts. Comprising a research team with seasoned and emerging youth researchers and having the support of established institutions can be considered pre-conditions for success. Factors of success include the meaningful involvement of young people in the research methods and process, fostering a positive working culture to shift power and having a flexible approach and budget which allows for training, knowledge exchange and mutual support.

While the critical factors for success have been documented for other youth participatory research approaches, the essential preconditions are often taken for granted [11, 24, 25]. However, studies about youth participation have shown that mentorship structures and institutional backing are crucial to sustain youth engagement beyond tokenistic roles [24, 25]. Additionally, our reflections contribute to emerging evidence that co-creating research activities enables youth researchers to take on leadership roles rather than only participatory or consulting ones, thereby building more equitable collaborations [22, 25].

These experiences demonstrate that research processes led by young people have clear added value, from enhancing the study relevance, capacitating youth researchers in their skills and professional development, to challenging what is considered ‘real research’ [3, 4]. Other research has found that youth involvement in research (such as youth participatory action research) has improved uptake of study findings [26]. As such, our findings show that youth-led research is subversive in its set-up by shifting power and challenging the status quo on who generates knowledge and who is considered an ‘expert’.

The focus on youth-led research may however conceal the fact that youth are not a homogenous group. Youth-led research should therefore not stop at involving young people contingent to their age, but should be mindful of their intersectionality with gender, ethnicity, ability, class, and more [20, 27], for example to inform team composition and collaboration dynamics. Given that young people are often in the beginning stages of their careers [3], it is important to ensure a mix of skill sets and skill levels in the research team, have a clear training trajectory to strengthen capacities, and account for sufficient time and flexibility for mutual mentoring and knowledge exchange. In this way, our lessons on team composition and mutual mentoring contribute new empirical evidence on how youth can mutually enhance youth leadership. This approach may not be suited to all topics (such as sexual and gender-based violence) or all contexts (such as humanitarian settings). However, the offer of equity and participation underlying youth-led research can be transformative for young people involved and the research process.

Taken together, our five lessons largely reinforce emerging literature on youth-led and youth-participatory action research but add nuance on how these approaches can be operationalised in practice. Specifically, we show that capacity-building and institutional support should be seen as foundational rather than supplementary; flexibility and trust are prerequisites for youth leadership; and attention to diversity within youth teams is crucial for equitable collaboration. Future research should further explore how these factors interact across different contexts, and under what conditions youth-led research can sustainably influence policy and practice.

## Data Availability

Data are available from the corresponding author upon reasonable request.
